# Integrative Multi-Omics Analysis Reveals the Immunoregulatory Effects of Sepia Ink on ADHD-like Phenotypes

**DOI:** 10.3390/cimb48040410

**Published:** 2026-04-16

**Authors:** Baohong Wei, Jiayi Yin, Wenmin Yuan, Peiling Cai, Qiaoling Song, Zhe Li, Xiaoqing Ma, Xue Yang, Lejia Hong, Huashi Guan, Guanhua Du, Wenzhe Yang

**Affiliations:** 1 Key Laboratory of Marine Drugs of Ministry of Education, School of Medicine and Pharmacy, Ocean University of China, Qingdao 266100, China; 2 Marine Biomedical Research Institute of Qingdao, Qingdao 266100, China

**Keywords:** sepia ink, ADHD, behavioral improvement, neuroinflammation, immunoregulation

## Abstract

Attention-Deficit/Hyperactivity Disorder (ADHD), affecting 5–10% of children globally, faces treatment limitations due to adverse effects and uncertain long-term risks of current pharmacotherapies. This study investigated the therapeutic potential of sepia ink (SI), a marine-derived natural complex from cuttlefish, in a scopolamine-induced ADHD-like mouse model. The chemical constituents of SI were characterized via Ultra-Performance Liquid Chromatography-Mass Spectrometry (UPLC-MS). The behavioral assessments, histopathological examinations, flow cytometry, and complete blood counts were utilized to evaluate its effects on ADHD-like phenotypes, neuroinflammation, and immune function. Integrated transcriptomic, plasma metabolomic, and 16S rRNA sequencing were used to explore the underlying mechanisms. SI significantly alleviated hyperactivity and improved spatial learning and memory deficits. It reduced hippocampal neuronal damage, attenuated neuroinflammation, and reversed scopolamine-induced immunosuppression in spleen and thymus. SI also restored the balance of immune cell subsets in both mesenteric lymph nodes and spleen, and the peripheral blood cell counts. Multi-omics analyses suggested that the beneficial effects of SI were associated with reduced neuroinflammation, rebalanced systemic immune responses, partial correction of lipid metabolic disturbances, and restoration of gut microbiota homeostasis. Collectively, our findings indicate that SI effectively mitigates the in vivo ADHD-like impairments by coordinating immune, metabolic, and gut microbiota-related processes, thereby supporting its potential as a marine-derived therapeutic candidate for further ADHD treatment.

## 1. Introduction

ADHD is a prevalent neurodevelopmental disorder characterized by persistent patterns of inattention, hyperactivity, and impulsivity that interfere with daily functioning and development [1]. It affects approximately 5–10% of children worldwide and often persists in adulthood [2]. The clinical manifestations of ADHD include difficulties in sustaining attention, excessive motor activity, and impulsive decision-making, which can lead to academic underachievement, social challenges, and emotional dysregulation [3]. The etiology of ADHD is multifactorial, involving a complex interplay of genetic, environmental, and neurobiological factors [4]. Dysregulation of neurotransmitter systems, particularly dopamine and norepinephrine, has been implicated in the pathophysiology of ADHD [4]. Current first-line pharmacotherapies for ADHD rely on central nervous system (CNS) stimulants (e.g., methylphenidate, amphetamines) and non-stimulants (e.g., atomoxetine), yet their clinical applications face significant limitations. Specifically, stimulants carry abuse liability and provoke adverse cardiometabolic/neuropsychiatric effects [5]. The non-stimulants exhibit a delayed therapeutic onset with insufficient longitudinal safety data regarding neurodevelopmental impacts of prolonged pediatric exposure [6]. Non-pharmacological interventions are frequently hindered by limited accessibility and a high dependence on patient adherence [7]. Given these unresolved therapeutic challenges, it becomes a critical imperative to develop novel agents with high efficiency and favorable safety profiles.

Natural products remain an important source for modern drug discovery, and the marine environment has emerged as a rich reservoir of pharmacologically active compounds derived from diverse marine organisms [8]. To date, various marine-derived compounds or products have shown therapeutic potential, and numerous candidates are currently advancing through preclinical and clinical development pipelines [9]. In the field of neurological disorders, several marine-derived substances have attracted attention because of their favorable pharmacological properties. For example, marine cyanobacterial peptides demonstrate superior blood–brain barrier permeability [9], whereas polysaccharides such as fucoidan have been reported to exhibit multi-target neuromodulation, simultaneously inhibiting pro-inflammatory TNF-α/NF-κB pathways and activating neuroprotective BDNF/TrkB signaling [10]. These findings support the potential of marine natural products as a valuable resource for the development of new therapeutics for complex neurological diseases.

Sepia ink (SI), a marine-derived substance isolated from the ink sac of cuttlefish, has been reported to be rich in melanin [11], amino acids [12], dopamine [13], and other putative bioactive constituents. Previous studies have revealed the unique neurotherapeutic mechanisms of these components. For instance, melanin attenuates neuroinflammation, cognitive deficits, and sleep disturbances induced by influenza A virus (IAV) through modulation of the JNK/ERK signaling pathway, and reduction in pro-inflammatory cytokine levels [14], positioning it as a promising marine-derived therapeutic alternative for neurological disorders. Orally administered melanin ameliorated depression- and anxiety-like behaviors in Dextran sulfate sodium (DSS)-induced colitis mice [15,16]. While these observations suggest that SI may possess biological activities relevant to neurological disorders, its specific therapeutic potential for ADHD remains largely unexplored. Furthermore, its effects on the gut–brain axis and metabolic homeostasis in ADHD models have not been systematically investigated.

To bridge the aforementioned knowledge gaps, this study systematically evaluates the ameliorative effects of SI on scopolamine-induced ADHD-like phenotypes and explores the underlying mechanisms. By integrating UPLC-MS profiling with network pharmacology analysis, we characterized the chemical constituents of SI and mapped its putative target-pathway network. Subsequently, the in vivo validation confirmed that SI could ameliorate the ADHD-like phenotypes, including behavioral impairments and immune dysfunction in a scopolamine-induced mouse model. Furthermore, we utilized multi-omics technologies, including transcriptomics, plasma metabolomics, and 16S rRNA sequencing, to explore the molecular, metabolic, and gut-microbiota modulations associated with SI. This integrative approach illustrates SI as a promising marine-derived therapeutic regimen for ADHD and also provides insights into its systemic neuro-immunomodulatory effects, paving the way for its future clinical translation.

## 2. Materials and Methods

### 2.1. Materials

The Morris water maze was paired with the Smart3.0 software system, and the ZH-YLS-1C spontaneous locomotion recorder (provided by the Marine Biomedical Research Institute of Qingdao, Qingdao, China) was employed to assess the spontaneous activity of mice.

SI (Batch No. SI-231101) was prepared by the Marine Biomedical Research Institute of Qingdao. Atomoxetine hydrochloride (Cat. No. PHR1679, Sigma, St. Louis, MO, USA) was prepared as a solution with a concentration of 8.04 mg/mL. All samples were dissolved or mixed in 3% CMC-Na (Cat. No. MB1717, Meilunbio, Dalian, China). Scopolamine hydrobromide (Cat. No. S729115, Macklin, Shanghai, China) was purchased and stored under dry conditions at room temperature.

### 2.2. Analysis of Inorganic Elements

The SI samples were digested with HNO3 using a microwave digestion system (TOPEX, SINEO, Shanghai, China). The elements of Calcium (Ca) and magnesium (Mg) were determined by ICP-OES (PerkinElmer Optima 8000, PerkinElmer, Shelton, CT, USA). Trace elements, including manganese (Mn), iron (Fe), zinc (Zn), and selenium (Se), were quantified by ICP-MS (Thermo iCAP RQ, Thermo Scientific, Waltham, MA, USA). All elements were analyzed using external standard calibration curves.

### 2.3. UPLC-Q-Exactive Plus MS Analysis

#### 2.3.1. Chromatographic Separation

Firstly, LC analysis was performed on a SHIMADZU-LC30 (Shimadzu Corporation, Kyoto, Japan) ultra-high-performance liquid chromatography (UHPLC) system with a binary pump, an autosampler, and a column oven. The chromatographic separation was performed with an ACQUITY UPLC^®^ HSS T3 column (2.1 × 100 mm, 1.8 μm) (Waters, Milford, MA, USA). The injection volume was 10 μL, the column temperature was maintained at 40 °C, and the flow rate was 0.3 mL/min. The mobile phases consisted of 0.1% formic acid aqueous solution (A) and acetonitrile (B). The elution gradient was set as follows: 0–1 min, 2% (B); 1–5 min, 2–48% (B); 5–7 min, 48–80% (B); 7–11 min, 80–100% (B); 11–13 min, 100% (B); 13–13.01 min, 2% (B); 13.01–15 min, 2% (B).

#### 2.3.2. Mass Spectrometry Acquisition

Each sample was detected in positive (+) and negative (−) ion modes via electrospray ionization (ESI). After UPLC separation, samples were analyzed using a QE Plus mass spectrometer (Thermo Scientific, Waltham, MA, USA) with ionization achieved using a HESI source. The ionization conditions were set as follows: Spray Voltage: 3.8 kV (+) and 3.2 kV (−); Capillary Temperature: 320 °C (±); Sheath Gas: 30 (±); Aux Gas: 5 (±); Probe Heater Temperature: 350 °C (±); S-Lens RF Level: 50.

### 2.4. Animal Model and Experimental Design

SPF-grade male KM mice (3 weeks old, 12–14 g) were purchased from Jinan Pengyue Experimental Animal Breeding Co., Ltd. (Jinan, China, license number: SCXK(Lu)20220006). The mice were housed under controlled conditions (22 ± 2 °C, 50 ± 5% humidity) with a 12 h light/dark cycle. After a 7-day acclimatization period, mice were randomly divided into five groups: (1) Control group (Con): received 3% CMC-Na (i.g.); (2) Model group (Mod): received 3% CMC-Na (i.g.) followed by scopolamine hydrobromide (3 mg/kg, i.p.) to induce ADHD-like phenotype [17]; (3) Positive control group (Ato): received atomoxetine hydrochloride (8.03 mg/kg, i.g.) and scopolamine hydrobromide (3 mg/kg, i.p.); (4) Low-dose SI group (SIL): received SI (100 mg/kg, i.g.) and scopolamine hydrobromide (3 mg/kg, i.p.); (5) High-dose SI group (SIH): received SI (500 mg/kg, i.g.) and scopolamine hydrobromide (3 mg/kg, i.p.). Group allocation was performed using a concealed assignment procedure before treatment initiation.

The dose of atomoxetine hydrochloride was calculated by body surface area conversion from the clinical starting dose of atomoxetine and adjusted for the hydrochloride salt form. Scopolamine was administered on days 0, 22 and 25–29 while SI was given daily by oral gavage. All protocols in this study were approved by the Marine Biomedical Research Institute of Qingdao (approval number: E-MBWZ-2024-6-23).

### 2.5. Behavioral Tests

#### 2.5.1. Spontaneous Activity Test

On the 22nd day, the spontaneous activity of the mice was measured by a ZH-YLS-1C autonomous activity meter. Following the intraperitoneal injection of scopolamine, the mice were positioned in the spontaneous activity devices to adapt for 3 min. Subsequently, the number of spontaneous activities within 5 min was meticulously recorded. All tests and subsequent data acquisition were performed in a blinded fashion. Data were analyzed using one-way ANOVA followed by Bonferroni correction.

#### 2.5.2. Morris Water Maze Test

During the days 25–29 of administration, the Morris water maze test was performed according to a previous method [18]. The maze was composed of a circular pool with a diameter of 2.0 m, filled with water maintained at a temperature between 21 and 22 °C, and evenly partitioned into four distinct quadrants. A submerged, invisible platform was strategically placed within one of the quadrants. All tests and subsequent data acquisition were performed in a blinded fashion. To accurately evaluate the spatial learning and memory capabilities of the mice, two key parameters were meticulously recorded: the number of times the mice crossed the area where the platform had previously been located, and the amount of time they spent swimming within the target quadrant. Data were analyzed using one-way ANOVA followed by Bonferroni correction.

### 2.6. Complete Blood Count

At the end of the experiment, all mice were fasted for 12 h with only deionized water. Subsequently, mice were euthanized in parallel by carbon dioxide (CO_2_) inhalation. All abdominal aortic blood samples were collected in each group and placed in EP tubes coated with heparin sodium. 60 μL of whole blood was collected and analyzed using a ProCyte Dx Veterinary Hematology Analyzer (IDEXX Laboratories, Inc., Westbrook, ME, USA) with the corresponding ProCyte Stain Pack (Cat. No. 98-71001-00, IDEXX) and PDX Reagent Kit (Cat. No. 99-26306-00, IDEXX). The remaining blood samples were centrifuged at 3500 rpm for 15 min at 4 °C to separate the plasma. Data were analyzed using one-way ANOVA followed by Bonferroni correction.

### 2.7. Flow Cytometry

Mesenteric lymph nodes (MLNs) and spleens were harvested from each mouse and placed in PBS containing 2% fetal bovine serum (FBS). Tissues were gently ground and filtered through a 100 μm cell strainer to prepare cell suspensions; splenic suspensions were treated with red blood cell (RBC) lysis buffer (Cat. No. 130-094-183, Miltenyi Biotec, Bergisch Gladbach, Germany) to eliminate erythrocytes. Subsequently, the cell suspensions were centrifuged and resuspended in blocking buffer (containing 20% FBS, 1% immunoglobulin G (IgG, Cat. No. I5381, Sigma), and 1% CD16/CD32 antibody (Cat. No. 14016182, Thermo)). After blocking, the suspensions were incubated with fluorescent anti-mouse antibodies, followed by staining with 7-AAD (Cat. No. 420404, BioLegend, San Diego, CA, USA). Post-incubation, cells were washed with PBS, resuspended, and analyzed using a flow cytometer (BD FACSAria III, BD Biosciences, Franklin Lakes, NJ, USA). Single cells were gated using forward scatter height versus forward scatter area (FSC-H vs. FSC-A), and dead cells were excluded based on 7-AAD staining. The antibodies used were FITC anti-mouse CD4 (Cat. No. 100406, BioLegend), PE/Cyanine7 anti-mouse CD8a (Cat. No. 100722, BioLegend), and APC anti-mouse CD19 (Cat. No. 152409, BioLegend). The proportions of cell subsets were quantified with FlowJo software V10.8.1.4. Data were analyzed using one-way ANOVA followed by Bonferroni correction.

### 2.8. Histopathological Analysis

Upon euthanasia, multiple tissue samples were promptly collected. Tissues were carefully removed and immersed in 4% paraformaldehyde (*v*/*v*) for a 24 h fixation period. Subsequently, the fixed tissues were processed for paraffin embedding, which was then followed by hematoxylin and eosin (H&E) staining. The stained sections of the brain, spleen, and thymus were observed under an optical microscope (Olympus, Tokyo, Japan), and all observations were performed in a blinded manner.

### 2.9. RNA-Seq Analysis

The left brain was dissected from euthanized mice on ice, snap-frozen in liquid nitrogen, and total RNA was extracted using TRIzol. Libraries were constructed with rRNA depletion and sequenced on an Illumina NovaSeq 6000 (Illumina, San Diego, CA, USA, 150 bp paired-end reads). Raw reads were filtered using Trimmomatic (v0.39) to remove adapters, low-quality reads (Q-value < 20), and reads containing ambiguous bases. Gene expression levels were quantified as fragments per kilobase of transcript per million mapped reads (FPKM) using StringTie (version 3.0.3). Differentially expressed genes (DEGs) were identified using DESeq2 with thresholds of fold change (FC) > 1.5 and *p*-value < 0.05. Functional enrichment analysis of DEGs, including Gene Ontology (GO) and Kyoto Encyclopedia of Genes and Genomes (KEGG) pathway analyses, was performed using clusterProfiler (version 4.16.2) in R software (version 4.4.0). A protein–protein Interaction (PPI) network was constructed using STRING (v11.5, confidence > 0.7), visualized via Cytoscape (v3.9.1). Gene set enrichment analysis (GSEA) was performed on genes ranked by log2FC to identify enriched Hallmark/KEGG/GO sets. IPA (QIAGEN) analyzed DEGs for pathway enrichment, upstream regulators, and disease/function, with significant canonical pathways defined by *p* < 0.05 and activation z-score > |2|.

### 2.10. Plasma Untargeted Metabolomics Analysis

For each mouse, 200 µL of plasma obtained in Section 2.6 Complete blood count was used and added to an 800 µL mixture of cold methanol and acetonitrile (1:4). After 5.0 min, the miscible liquids were centrifuged at 4000 rpm for 10.0 min to obtain the supernatants. All supernatants were rapidly dried with nitrogen and stored in a refrigerator at −80 °C until use. Additionally, 10 µL from each plasma sample was pooled to create quality control (QC) samples, which were used to monitor the instrument’s stability after every five plasma samples.

Untargeted metabolomic analysis was performed using UHPLC-MS/MS on a Q-Exactive system (Thermo Fisher Scientific) equipped with an ESI source. Chromatographic separation was achieved on an ACQUITY UPLC BEH C18 column (150 mm × 2.1 mm, 1.7 µm) at 35 °C, with mobile phases consisting of 0.1% formic acid in water (A) and acetonitrile (B) at a flow rate of 0.3 mL/min. Injection volume was 3 μL. Mass spectrometry conditions were set as follows: ion spray voltages of 3.5 kV (positive mode) and 3.0 kV (negative mode); sheath gas flow 30 arb, auxiliary gas flow 10 arb; capillary temperature 325 °C, auxiliary gas heater temperature 350 °C; full scan range *m*/*z* 70–1050. Data were acquired in Full MS/dd-MS2 mode with resolutions of 70,000 (MS) and 35,000 (MS/MS).

Raw data were processed using Compound Discoverer 3.0 (Thermo Fisher) for noise reduction, baseline correction, and normalization, with parameters set for elemental composition (C 0–60, H 0–120, O 0–10, N 0–4, P 0–3, S 0–3) and RDB 0–15 (mass accuracy < 5 ppm). Multivariate analyses were conducted using SIMCA-P 14.0. Both PCA and OPLS-DA were conducted after Pareto scaling. PCA was used primarily to visualize overall distribution trends, with a 95% confidence interval, and group differences were assessed using analysis of similarities (ANOSIM). OPLS-DA was used to further examine group separation, and model overfitting was evaluated by 200 permutation tests; a Q2 value > 0.5 was considered indicative of good predictive performance. Differential metabolites were screened based on a combined assessment of S-plots, variable importance in projection (VIP) scores from OPLS-DA, and univariate statistical results. Univariate analysis was performed using an unpaired two-tailed Student’s *t*-test.

### 2.11. 16S rRNA Gene-Based Amplicon Sequencing and Bioinformatic Analysis

Fecal genomic DNA from the Con, Mod, and SIH groups was extracted for 16S rRNA gene analysis. Libraries were constructed using the NEB Next^®^ Ultra DNA Library Prep Kit (Illumina) with index labeling and quality-checked on an Agilent 5400 system. Sequencing was performed on an Illumina platform to generate 250 bp paired-end reads.

Bioinformatics analysis was performed in QIIME2 following the Atacama soil microbiome tutorial with custom scripts [19]. Raw FASTQ data were processed via the dada2 plugin for quality filtering, trimming, denoising, merging, and chimera removal to obtain amplicon sequence variant (ASV) tables. Taxonomic classification was assigned using the feature-classifier plugin against a pretrained reference database, followed by removal of contaminating sequences. For alpha diversity, group comparisons were performed using one-way ANOVA with FDR correction for multiple testing, followed by Games-Howell post hoc testing. For beta diversity, principal coordinates analysis (PCoA) and non-metric multidimensional scaling (NMDS) were both based on the Bray–Curtis distance metric, and group differences were assessed using analysis of similarities (ANOSIM) with 999 permutations. Differential taxa were identified using LEfSe, with thresholds of LDA > 2 and *p* < 0.05, across taxonomic levels from kingdom to genus. Genus-level differences shown in the Venn diagram were based on the absolute abundance table analyzed by DESeq, with a minimum feature prevalence of 0.2 and screening criteria of log2FC > 2 and *p* < 0.05. The stacked bar plot displays the top 20 most abundant genera.

### 2.12. Statistical Analysis

Data analysis was conducted using Statistical Product and Service Solutions (SPSS) 22.0 software, with results expressed as mean ± standard error of the mean (SEM). Statistical significance for group comparisons was evaluated by one-way analysis of variance (ANOVA), where *p* < 0.05 was considered statistically significant. Graphical representations and statistical outputs were generated using GraphPad Prism 9.0 software.

## 3. Results

### 3.1. Identification of SI Components and Prediction of Their Therapeutic Potential in ADHD

**Figure 1:**
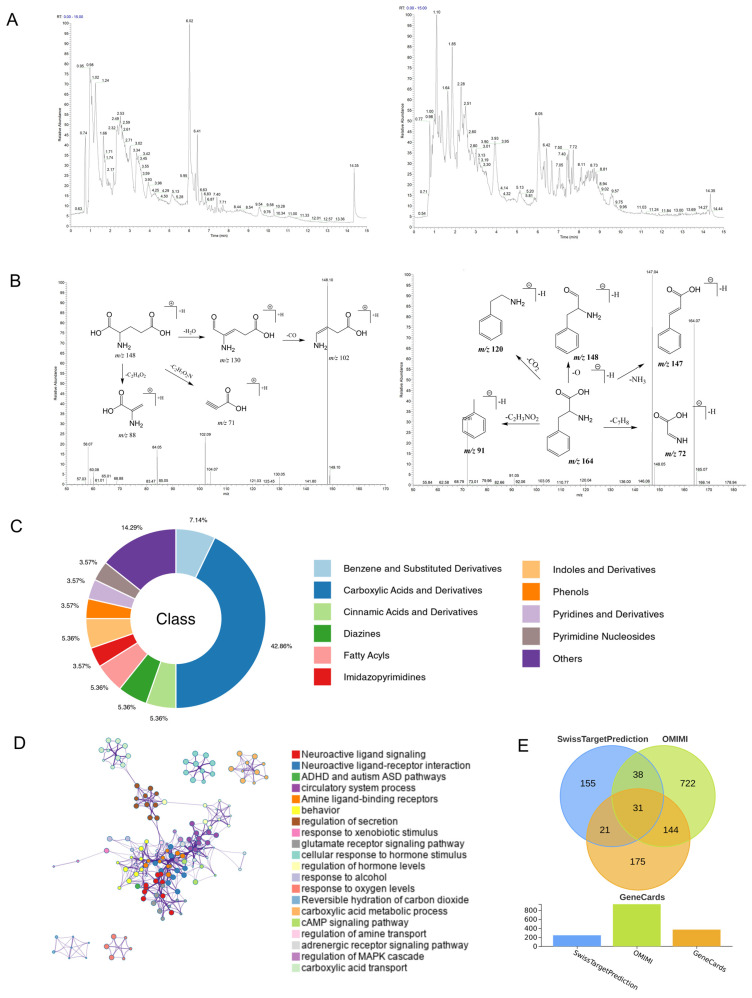
Component identification and target prediction of SI. (**A**) Total ion chromatograms (TIC) of SI components obtained by UPLC-Q-Exactive Plus MS analysis in positive (left) and negative (right) ionization modes. (**B**) Representative MS/MS fragmentation spectra of two of the key compounds: Glutamic acid (left) and Phenylalanine (right). (**C**) Classification of 56 identified components in SI. (**D**) Functional enrichment analysis of the predicted targets for SI components. (**E**) Venn diagram of the intersection of targets.

The chemical constituents of SI were comprehensively characterized using UPLC-Q-Exactive Plus MS. The total ion chromatograms (TIC) under both positive and negative ionization modes are presented in Figure 1A. A total of 245 distinct components were successfully identified based on their MS and MS/MS spectra. After excluding non-marine-derived compounds using CHEMnetBASE (https://dmnp.chemnetbase.com/, accessed on 2 April 2026) [20], 56 compounds were retained and categorized into several major classes, including carboxylic acids and derivatives, and so on (Figure 1C). These components were further subjected to target prediction using the SwissTargetPrediction (www.swisstargetprediction.ch, accessed on 2 April 2026) [21]. Subsequent functional enrichment analysis of the putative targets (probability > 0.1) was performed via Metascape (www.metascape.org/, accessed on 2 April 2026) [22]. The results revealed a significant enrichment of pathways directly relevant to ADHD, including “Neuroactive ligand signaling”, “ADHD and autism ASD (Autism Spectrum Disorder) pathways” and “behavior” (Figure 1D). This enrichment pattern underscores the targeted therapeutic potential of SI components in ADHD.

Furthermore, the above candidate targets were intersected with ADHD-related targets retrieved from OMIM (https://omim.org/, accessed on 2 April 2026) [23] and GeneCards (www.genecards.org/, accessed on 2 April 2026) [24]. The 31 common targets were identified as potential therapeutic targets of SI in ADHD (Figure 1E). When the SI constituents were ranked by the number of overlapping targets, the top 10 compounds were identified and are listed in Table 1. The MS/MS spectra of two representative components, Glutamic acid and Phenylalanine, are shown in Figure 1B. Notably, several of these compounds have been reported to exhibit anti-inflammatory and neuroprotective activities. For instance, Oleamide significantly inhibited nitrite production and Prostaglandin E2 (PGE2) secretion, reduced the expression of Inducible Nitric Oxide Synthase (iNOS) and Cyclooxygenase-2 (COX-2), and suppressed the production of pro-inflammatory cytokines such as Tumor Necrosis Factor-alpha (TNF-α), Interleukin-1 beta (IL-1β), and Interleukin-6 (IL-6) in LPS-induced RAW264.7 murine macrophages [25]. Octopamine could drive the transformation of astrocytes from a toxic to a neuroprotective state by promoting aerobic glycolysis [26]. Furthermore, supplementation with the fatty acid amide (FAM) oleamide (ODA) in breast milk enhanced learning and memory in lactating mice [27].

In addition to its small-molecule constituents, SI is also rich in metal elements, which may contribute to its immunomodulatory potential. Quantitative analysis showed that SI contains Ca, Mg, Mn, Fe, Zn, and Se (Table 2). Previous studies have shown that zinc deficiency can impair both innate and adaptive immunity, and chronic deficiency may further promote inflammatory responses [28]. In addition, magnesium has been reported to regulate the effector function of CD8+ T cells through LFA-1 (Lymphocyte Function-associated Antigen 1) [29]. These findings suggest the immunomodulatory potential of SI based on its inorganic elemental composition. In summary, these findings suggest that the bioactive constituents of SI might synergistically mitigate the ADHD-like phenotypes by coordinating the anti-inflammatory signaling, immune cell regulation, and neuromodulation.

### 3.2. SI Alleviated Scopolamine-Induced Hyperactivity and Memory Impairment In Vivo

**Figure 2:**
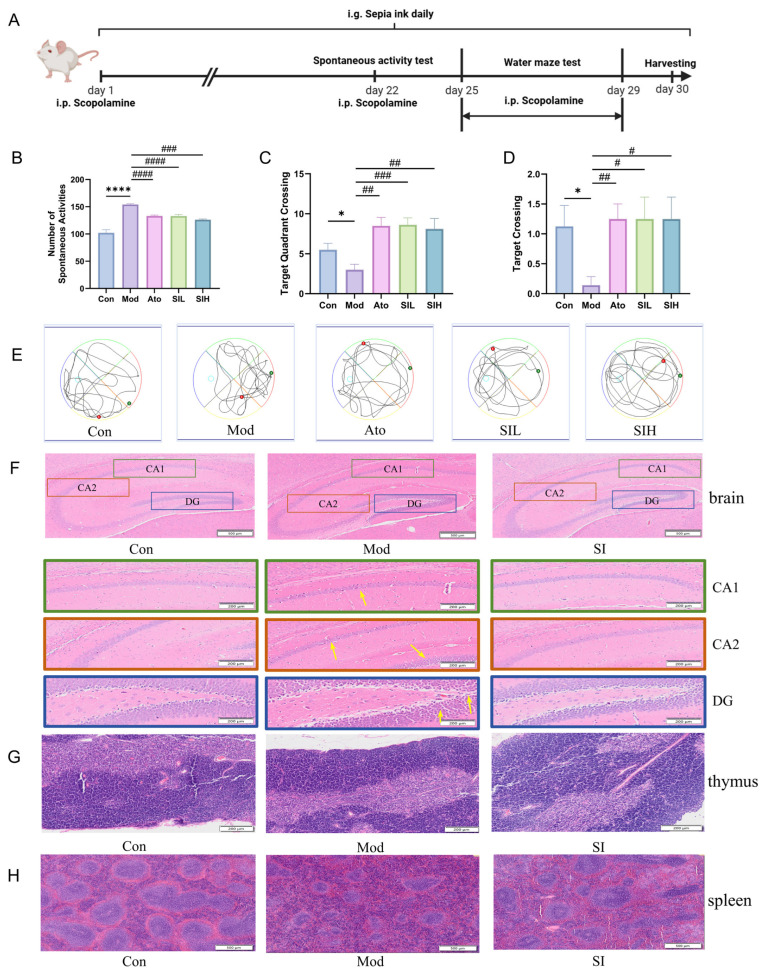
SI alleviated scopolamine-induced ADHD-like behavioral abnormalities and associated histopathological damage. (**A**) The procedures for model establishment, drug administration, and various experimental testing protocols in experimental mice. On days when scopolamine and SI were both administered, SI was given 6 h after scopolamine injection. (**B**) The number of spontaneous activities (*n* = 12). (**C**) The target quadrant time (*n* = 8). (**D**) The platform-crossing times of target quadrant (*n* = 8). (**E**) The swim traces on the spatial probe trial. Green indicates the “begin” point, red indicates the “end” point, and blue indicates the target platform. (**F**) H&E staining of brain hippocampus (500 μm), CA1, CA2 and DG (200 μm) in groups. The yellow arrow indicates loose neuronal connections. (**G**) H&E staining of thymus (scale bar, 200 μm) in groups. (**H**) H&E staining of spleen (scale bar, 500 μm) tissues in groups. Data are expressed as the means ± SEM, * *p* < 0.05, **** *p* < 0.0001. Mod_vs_Con; # *p* < 0.05, ## *p* < 0.01, ### *p* < 0.001, #### *p* < 0.0001. SI_vs_Mod.

Scopolamine can induce behavioral and neurobiological alterations relevant to hyperactivity and cognitive impairment in mice [30,31], and was therefore used in the present study as a pharmacological approach to model ADHD-like phenotypes in rodents [32]. To evaluate the therapeutic potential of SI, mice were randomly divided into five groups: Control (Con), Model (Mod), Positive control (Atomoxetine, Ato), Low-dose SI (SIL), and High-dose SI (SIH). As shown in Figure 2A, scopolamine was administered on days 0, 22, and 25–29, while SI was given daily by oral gavage. On days of co-treatment, SI was administered 6 h after scopolamine injection. The spontaneous activity test revealed a substantial increase in spontaneous activity in the Mod group compared to the Con group (*p* < 0.01), confirming the successful induction of hyperactivity-like behavior. SI treatment at either low or high dose (SIL and SIH) significantly attenuates these excessive movements (Figure 2B). The beneficial effects of SI on ADHD-like phenotypes were also observed in SHR rats. In the open field test, SI treatment partially normalized the increased locomotor activity and altered exploratory behavior seen in the Mod group (Figure S1).

To further explore the impact of SI on scopolamine-induced cognitive impairment, we subjected the mice to the Morris water maze task. The target quadrant time of the Mod group was significantly lower than that of the Con group (*p* < 0.05), reflecting impaired spatial memory. The Ato, SIL, and SIH groups exhibited prolonged target quadrant times, with SIH showing the most pronounced effects, indicating the effective rescue of spatial memory (Figure 2C). As shown in Figure 2D, the Mod group displayed reduced platform-crossing frequencies, further confirming the memory impairment. The Ato, SIL, and SIH groups increased the crossing frequencies, with SIH showing a more pronounced effect, suggesting that SI at high dose (SIH) is more effective in enhancing memory-related behavior. Moreover, the Mod group exhibited random search trajectories, while both the Con group and treatment groups (Ato, SIL, and SIH) displayed highly localized swim paths in the target quadrant. These data confirmed that SI, as well as Ato, could restore the spatial memory retention (Figure 2E). Similarly, in the Morris water maze probe trial, SI improved target quadrant preference and crossing frequency (Figure S2). Together, these results indicate that SI effectively alleviates cognitive impairment in ADHD-like phenotypes in vivo.

Consistent with the behavioral findings, H&E staining of the hippocampus revealed histopathological alterations in the Mod group. Compared with the normal control group, the model group showed reduced neuronal numbers and cellular atrophy in the Cornu Ammonis 1 (CA1) and CA2 regions, together with a more eosinophilic appearance, suggesting pathological alterations in these areas. After SI treatment, neuronal atrophy was alleviated (Figure 2F). In the Dentate Gyrus (DG) region, the model group exhibited loosely arranged neurons with obvious intercellular spaces, which may indicate weakened neuronal connectivity. These histopathological changes were improved after SI administration (Figure 2F).

### 3.3. SI Restored Immune Homeostasis in Scopolamine-Induced ADHD-like Mouse Model

As mentioned in Table 2, the metal elements of SI linked to ADHD possess potential immune-regulatory effects. Next, a comprehensive evaluation was conducted of the key lymphoid organs, including the thymus, spleen, and mesenteric lymph nodes (MLNs), as well as peripheral blood counts. The HE staining of the thymus revealed that the model group had significantly fewer lymphocytes in the thymic cortical region, with a thinner cortex and reduced cell density, indicating suppressed immune response (Figure 2G). In the SI group, lymphocyte proliferation in the cortical region was observed, along with a thicker cortex and increased cell density, suggesting enhanced immune response (Figure 2G). Meanwhile, the model group showed disorganized splenic architecture, abnormal atrophy of lymphocytes in the white pulp with structural disorder, and a significantly increased proportion of red pulp compared with the control group, indicating excessive immune suppression (Figure 2H). In the SI-treated group, the architecture of the spleen was partially restored, with significant lymphocyte proliferation in the white pulp, partial recovery of white pulp structure, and clear demarcation between red and white pulp, suggesting improved immune response (Figure 2H). These pathological changes suggested that SI could reverse the immunosuppressive status of the key lymphoid organs.

**Figure 3:**
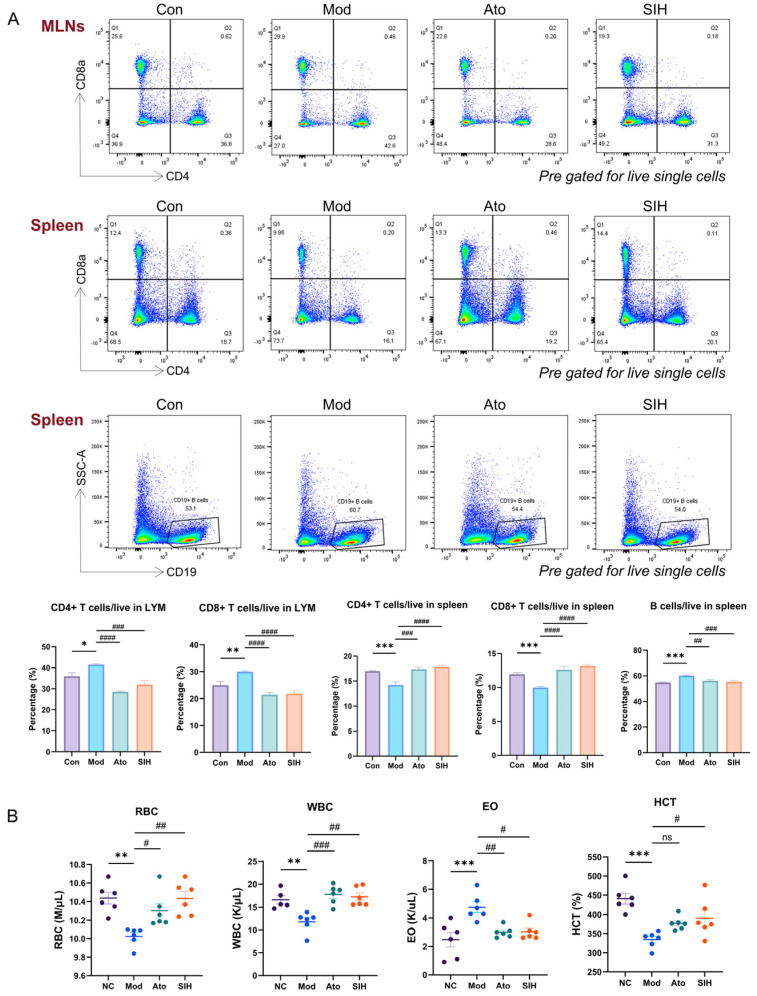
SI restored immune dysregulation in scopolamine-induced ADHD-like phenotypes. (**A**) Flow cytometry analysis of immune cell proportions in mesenteric lymph nodes (MLNs) and spleen (*n* = 6). (**B**) Complete blood count of peripheral blood (*n* = 6). Data are expressed as the means ± SEM, * *p* < 0.05, ** *p* < 0.01, *** *p* < 0.001. Mod_vs_Con; # *p* < 0.05, ## *p* < 0.01, ### *p* < 0.001, #### *p* < 0.0001. SI_vs_Mod.

Flow cytometry and complete blood count were further employed. In MLNs, the model group exhibited elevated proportions of CD4+ and CD8+ T cells, which were normalized by SI treatment. Conversely, in the spleen, the model showed decreased CD4+ and CD8+ T cells along with an increase in B cells, which were likewise reversed by SI (Figure 3A). Moreover, hematological parameters, including Red Blood Cell (RBC), White Blood Cell (WBC), Eosinophil (EO), and Hematocrit (HCT), were dysregulated in the model group and restored to normal levels following SI intervention (Figure 3B). Collectively, alongside the behavioral improvements and the alleviation of neuropathological damage, SI could robustly restore the immune homeostasis of critical lymphoid organs and peripheral blood counts disturbed by scopolamine.

### 3.4. Transcriptome Corroborated the Central Anti-Inflammatory and Immunomodulatory Effects of SI In Vivo

**Figure 4:**
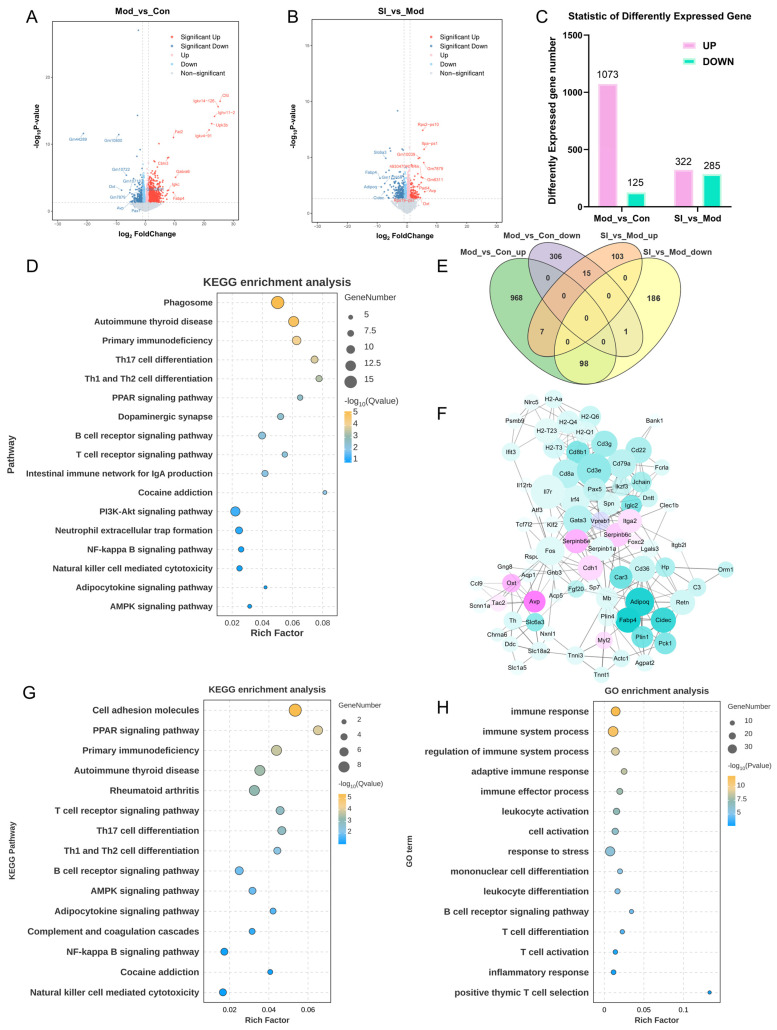
Transcriptomic analysis reveals SI-mediated immune and neuroinflammatory regulation in scopolamine-induced ADHD-like phenotypes. (**A**) Volcano plot of DEGs in Mod_vs_Con. Red/blue dots represent significantly up/downregulated genes (*p* < 0.05, FC > 1.5). (**B**) Volcano plot of DEGs in SI_vs_Mod. (**C**) Bar plot quantifying DEGs in Mod_vs_Con and SI_vs_Mod comparisons. (**D**) KEGG enrichment analysis of upregulated genes in Mod_vs_Con. (**E**) Venn diagram showing overlap of DEGs across comparisons. (**F**) Protein–Protein Interaction Network of DEGs regulated by SI. Green indicates downregulated genes, and pink indicates upregulated genes. (**G**,**H**) KEGG (**G**) and GO (**H**) enrichment analysis of the 98 overlapping genes that were upregulated in the Mod group and downregulated in the SI group.

To further explore the potential mechanisms underlying the effects of SI on ADHD-like phenotypes, brain tissues from mice in the control (Con), model (Mod), and high-dose SI (SI) groups were subjected to RNA-seq analysis. The differentially expressed genes (DEGs) were screened with a cutoff of fold change (FC) > 1.5 and *p* value < 0.05. Results showed that compared with the control, 1073 genes were significantly up-regulated and 125 genes were significantly down-regulated in the Mod group (Figure 4A,C). KEGG enrichment analysis of up-regulated DEGs indicated enrichment in inflammation and immune-related pathways, including Th17 cell differentiation, Th1/Th2 cell differentiation, and NF-κB signaling pathway (Figure 4D), suggesting that scopolamine-induced ADHD-like phenotypes were accompanied by activation of immune and inflammatory pathways. To further examine the molecular changes associated with SI treatment, gene expression changes between SI and Mod were compared, identifying 322 up-regulated and 285 down-regulated genes (Figure 4B,C). A protein–protein interaction (PPI) network was constructed based on these DEGs (Figure 4F), in which pink nodes represent genes up-regulated by SI and green nodes represent genes down-regulated by SI. Genes such as adiponectin (Adipoq) and peroxisome proliferator-activated receptor γ (Pparg) (related to fat metabolism), and immune-related genes such as H2-Aa and H2-DMb1 were enriched in the network.

Venn diagram analysis of the DEGs from the two comparison groups showed overlapping genes concentrated in “Mod_vs_Con_up” and “SI_vs_Mod_down”, with 98 genes up-regulated in Mod being down-regulated by SI (Figure 4E). KEGG enrichment analysis of these 98 genes showed significant enrichment in immune-related pathways, including “cell adhesion molecules”, “PPAR signaling pathway”, and “T cell receptor signaling”, suggesting SI intervention may be associated with coordinated regulation of immune-, metabolic-, and cell interaction-related processes (Figure 4G). GO enrichment analysis also highlighted terms such as “immune response”, “adaptive immune regulation”, and “lymphocyte activation”, supporting the possible involvement of immune-related mechanisms in the effects of SI (Figure 4H). These transcriptomic findings were broadly consistent with the immune-related changes observed in peripheral tissues, including thymus, spleen, and MLNs (Figure 2G,H). Together, the data suggest that SI treatment exhibits both central and peripheral immune-related modulation in the scopolamine-induced model, most possibly contributing to the mitigation of ADHD-like phenotypes.

### 3.5. Comparative Analysis of the Anti-Inflammatory and Immunomodulatory Effects of SI Using IPA

**Figure 5:**
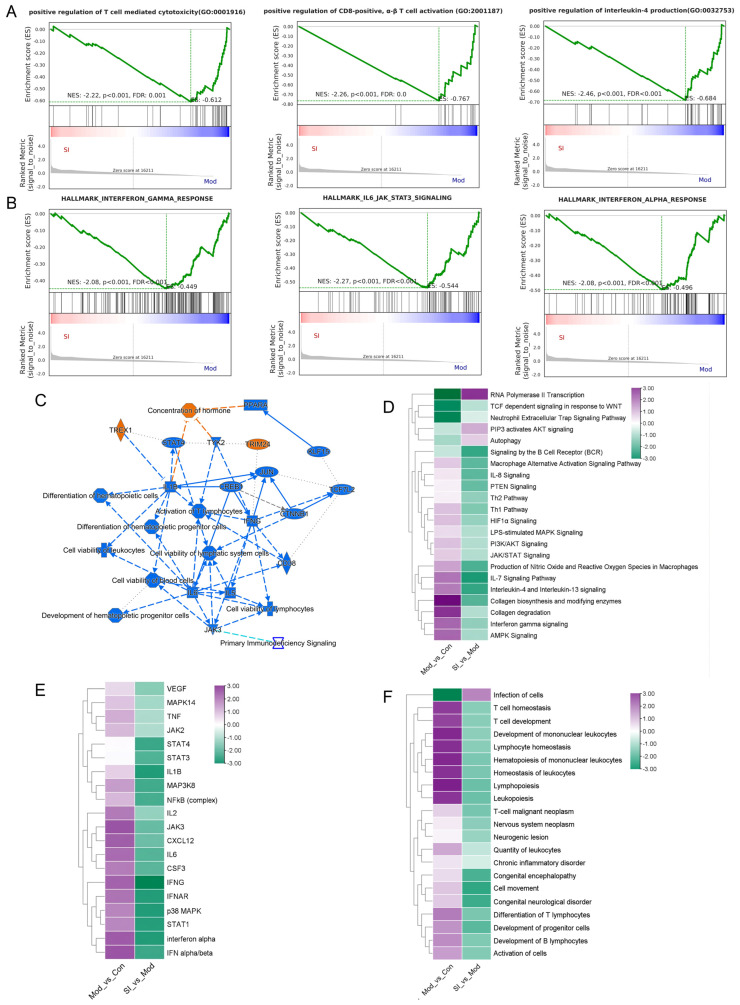
Comparative analysis of SI anti-inflammatory and immune regulation efficiency. (**A**) GSEA of immune-related terms. (**B**) GSEA analysis of inflammation-related terms. (**C**) Graph summary of SI_vs_Mod, blue nodes indicate inhibited pathways and orange nodes indicate pathways activated by SI. (**D**–**F**) Canonical Pathway Comparison (**D**), Upstream Regulator Comparison (**E**), Pathway & function Comparison (**F**) were conducted by comparing the significant differences between Mod_vs_NC and SI_vs_Mod. Positive z scores indicate an increase whereas negative ones indicate a reduction in specific terms.

Consistent with the above analysis, GSEA of the SI_vs_Mod comparison further suggested that SI treatment was associated with changes in inflammatory and immunoregulatory pathways. As shown in Figure 5A, SI downregulated the pathways, including positive regulation of T cell-mediated cytotoxicity, positive regulation of differentiation of CD8-positive αβ T cells, and positive regulation of interleukin-4 production, suggesting its modulatory effects on immune-related processes. Figure 5B further showed that SI was associated with reduced enrichment of inflammatory pathways such as interferon-γ response, IL6-JAK-STAT3 signaling, and interferon-α response.

To further clarify the immunoregulation and anti-inflammatory mechanisms of SI, we performed comparative analyses of Mod_vs_Con and SI_vs_Mod using IPA software (2025). In the summary graph (Figure 5C), the main interaction networks of SI are presented. At the canonical pathway level, pro-inflammatory pathways up-regulated in the model group, such as LPS-stimulated MAPK signaling and JAK/STAT signaling, showed reduced activation after SI treatment (Figure 5D). At the upstream regulatory level, factors predicted to be activated in the model group, including TNF, JAK2/3, STAT3/4, IL6, IL1B, CXCL12, and Interferon-γ (IFNG), were down-regulated after SI intervention (Figure 5E). At the disease and function level, IPA analysis suggested that SI treatment was associated with reduced activation of neurological diseases (e.g., neurogenic lesion, congenital neurological disorders) and inflammatory immune abnormalities (e.g., chronic inflammatory disorder, T cell development and differentiation of T lymphocytes) (Figure 5F). In conclusion, these transcriptomic analyses provide supportive evidence that SI treatment was associated with modulation of inflammation and immune-related molecular pathways in the brain tissue of the scopolamine-induced ADHD-like mouse model.

### 3.6. Plasma Metabolomics Reveals SI-Associated Changes in Lipid- and Immune-Related Metabolic Pathways in Scopolamine-Induced ADHD-like Phenotypes

**Figure 6:**
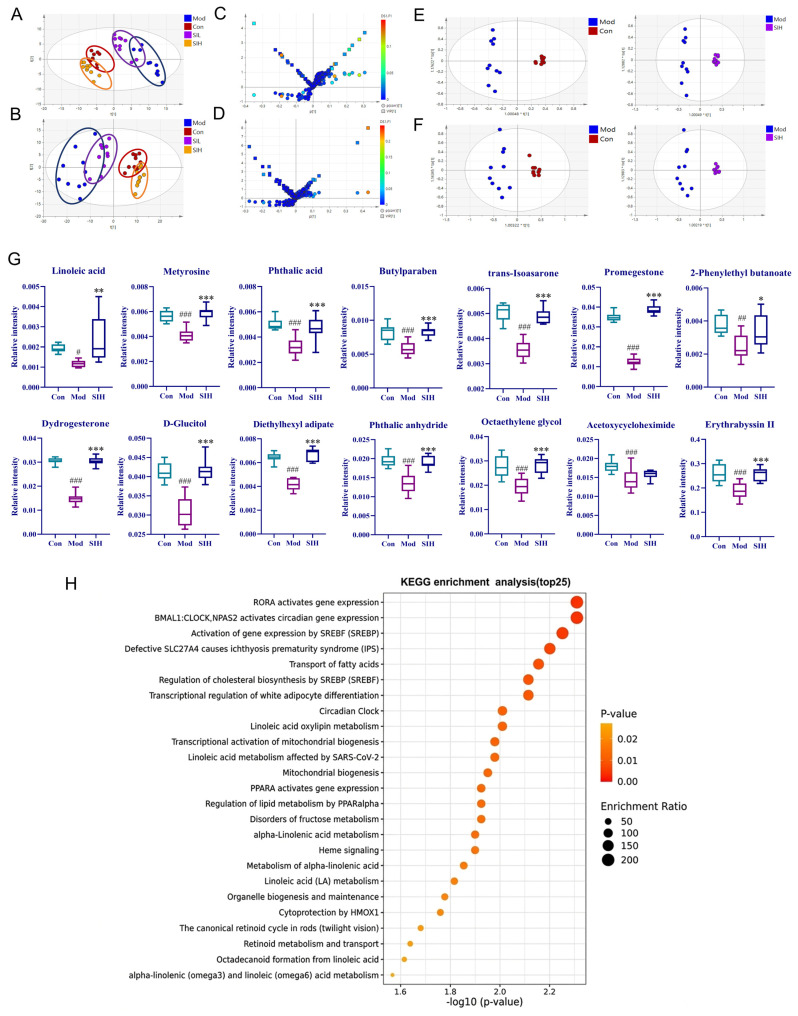
Plasma metabolomics analysis of SI-treated mice with scopolamine-induced ADHD-like phenotypes. (**A**,**B**) Plots of PCA scores. (**C**,**D**) S-plot and VIP plots of the Mod and SIH groups (**E**,**F**) OPLS-DA plots. (**G**) Results of 14 differential metabolites among the three groups. # *p* < 0.05. ## *p* < 0.01, and ### *p* < 0.001, Mod_vs_Con; * *p* < 0.05. ** *p* < 0.01, and *** *p* < 0.001, SIH_vs_Mod. (**H**) Top 25 KEGG-enriched pathways from the enrichment analysis of 14 differential metabolites in (**G**).

To elucidate the systemic influence of orally administered SI, plasma metabolomics was utilized to measure the circulating metabolic changes and decipher their potential contributions to the neuro-immunomodulation. LC-MS-based plasma metabolomic profiling in negative and positive ion modes showed distinct chromatographic patterns among groups (Figure S3). The model group deviated markedly from the control group in PCA space, suggesting metabolic disturbance after scopolamine treatment. Notably, high-dose SI (SIH) partially overlapped with the control group, indicating a trend toward metabolic restoration (Figure 6A,B). Similar group separation patterns were observed in the OPLS-DA analysis (Figure 6E,F). The differential metabolites were screened and identified by S-plot and VIP values, with thresholds of P|(corr)| > 0.4 and VIP > 1.0 (Figure 6C,D). Metabolites with statistically significant differences in relative abundance (*p* < 0.05) were identified as potential markers, and the HMDB database was used for metabolite annotation. A total of 14 metabolites changed significantly among the Con, Mod and SIH groups (Figure 6G).

To further characterize these changes, the differential metabolites were imported into MetaboAnalyst 5.0 software for KEGG pathway enrichment analysis (Figure 6H). The enriched pathways included RORA (RAR-related Orphan Receptor A) activating gene expression, Transport of fatty acids, Linoleic acid oxylipin metabolism, and Transcriptional activation of mitochondrial biogenesis in plasma. Emerging evidence suggests that the aforementioned metabolic pathways are intricately linked to immune regulation. Among them, RORA, a member of the nuclear receptor superfamily, has been implicated in the regulation of circadian rhythm, inflammation, metabolism, and cell development [33]. In RORA loxp/loxp Mx-1 Cre mice, significant increases in B cell populations were observed in peripheral blood, indicating its suppressive role in B cell proliferation and differentiation [34]. Furthermore, in vitro Parkinson’s disease models demonstrate that RORA agonists confer substantial neuroprotection through apoptosis inhibition and mitochondrial Reactive Oxygen Species (ROS) reduction [35]. In addition, fatty acid transport and turnover are increasingly recognized as being linked to immune and neurological functions [36,37,38]. Notably, several differential metabolites were functionally related to mitochondrial and redox-associated processes. Linoleic acid, which is involved in membrane structure and energy metabolism [39,40], was decreased in the model group and partially restored after SI treatment. Methyrosine, related to tyrosine hydroxylase and catecholamine biosynthesis [41], also showed altered levels. These changes suggest that SI may influence mitochondria-related metabolic processes. In summary, the metabolic pathway changes induced by high-dose SI intervention are closely associated with the immune response trend, and this multifaceted relationship may collectively contribute to the therapeutic effects of SI on scopolamine-induced ADHD-like phenotypes.

### 3.7. SI Modulated Gut Microbiota Profiles in Scopolamine-Induced ADHD-like Phenotypes

**Figure 7:**
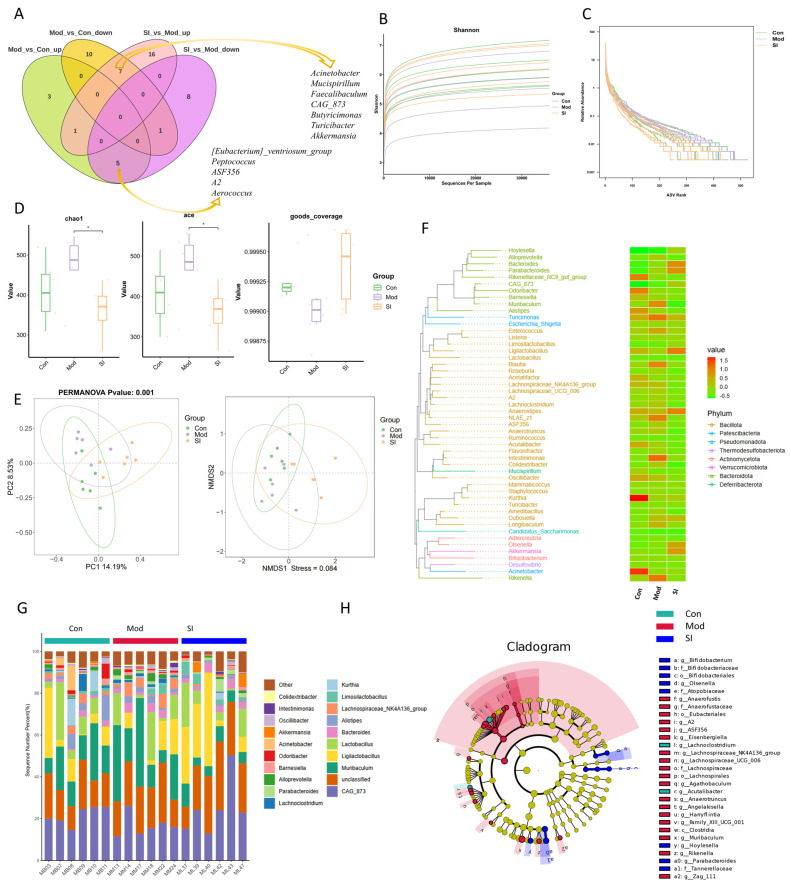
16S rRNA sequencing uncovers SI-mediated gut microbiota remodeling in scopolamine-induced ADHD-like phenotypes. (**A**) Venn Diagram of Differential ASVs. (**B**) Rarefaction curves of Shannon diversity index. (**C**) Rank abundance curves display species richness/evenness. (**D**) Boxplots of alpha diversity metrics (Chao1, ACE, goods_coverage). * *p* < 0.05. (**E**) Principal Coordinates Analysis (PCoA) and Non-metric Multidimensional Scaling (NMDS) of Bray–Curtis distances. (**F**) Heatmap of differentially abundant genera (row-wise) across samples (column-wise), clustered by Bray–Curtis distance. (**G**) Stacked bar plots of genus-level microbiota composition. (**H**) Linear discriminant analysis (LDA) effect size (LEfSe) cladogram identifying taxa differentially abundant across groups.

Next, changes in the gut microbiota were explored in SI-treated mice using 16S rRNA sequencing. In the α-diversity analysis, the box plot (Figure 7D) revealed a distinct pattern in ADHD-like phenotypes: elevated Chao1 and ACE indices indicating increased species richness, yet a reduced Shannon index (Figure 7B) reflecting decreased evenness. This pattern of increased richness with reduced evenness suggests that although the total number of species increased, their distribution became more uneven. This disorder aligns with reported intestinal microecological dysregulation in ADHD patients [42]. For the SI-treated group, Shannon, Chao1, and ACE gradually converged toward control levels. This suggests SI can reverse scopolamine-induced microbiota abnormalities. In the β-diversity analysis, samples in the Mod group deviated from the Con group along the PCoA and in NMDS dimensions (Figure 7E). While samples in the SI-treated group did not cluster closer to the Con group, they exhibited a distinct distribution compared to the Mod group, indicating that SI intervention can reshape the intestinal microbiota structure to some extent. Such structural modifications could be relevant, as a balanced microbiota structure is thought to support intestinal barrier integrity, immune function, and metabolic processes [43].

The Venn diagram (Figure 7A) shows the distribution of different amplicon sequence variants (ASVs) across groups. Cluster heatmaps (Figure 7F) and species composition analyses (Figure 7G) supported these observations: compared to the Mod group, the SI-treated group showed a trend toward decreased abundance of potentially harmful genera (e.g., Mucispirillum, a genus associated with chronic inflammation [44]) and increased abundance of beneficial taxa, including short-chain fatty acid (SCFA)-producing Akkermansia [45]. The cladogram (Figure 7H) visually demonstrated that SI treatment was associated with downregulation of pro-inflammatory genera (e.g., Acinetobacter [46]) that were enriched in the Mod group, alongside upregulation of beneficial genera such as Lactobacillus. These microbiota shifts were accompanied by potential functional implications: reduced abundance of pro-inflammatory genera may contribute to lower levels of inflammatory mediators (e.g., LPS, TNF-α), which could mitigate intestinal mucosal and neuroinflammatory responses [47]. Conversely, increased colonization of beneficial bacteria may promote SCFA (Short-Chain Fatty Acid) synthesis [48]. As key gut–brain signaling molecules, SCFAs have the potential to regulate neurotransmitter levels (e.g., serotonin), which may be relevant to the modulation of ADHD-related symptoms such as inattention and hyperactivity [49]. These findings suggest that SI treatment was associated with partial remodeling of scopolamine-induced gut microbiota dysbiosis, as reflected by changes in microbial diversity, community structure, and the relative abundance of specific bacterial genera.

## 4. Discussion

As a prevalent neurobehavioral condition in childhood, ADHD manifests as developmentally inappropriate inattention and hyperactivity/impulsivity. Its etiology involves complex genetic, environmental, and neurobiological factors [50]. Current clinical treatments face challenges in reconciling efficacy, safety, and patient-specific applicability [51]. This impasse underscores the critical need for developing multi-target/multi-dimensional intervention paradigms to transcend existing treatment constraints. Natural medicines, including Traditional Chinese Medicine, have garnered increasing attention in ADHD therapeutic development due to their multitarget mechanisms and favorable safety profiles. In the present study, we evaluated the effects of SI in a scopolamine-induced mouse model showing ADHD-like phenotypes. Our results suggest that SI improves behavioral abnormalities, potentially via mechanisms involving anti-inflammatory effects, immunomodulation, restoration of metabolic homeostasis, and remodeling of the gut microbiota.

Integrative chemical profiling and transcriptomics suggested that immunomodulation and neuroinflammation suppression may be involved in the effects of SI. Elevated pro-inflammatory cytokines such as IL-6 and TNF-α are reported in ADHD patients and animal models, correlating with symptom severity and dopaminergic dysfunction [52]. In the present study, transcriptomics showed that SI treatment was associated with reduced enrichment of pathways related to T-cell activation, including Th17 (T helper 17 cell) differentiation and T-cell receptor signaling, and inflammation, such as NF-κB and JAK-STAT pathways. Evidence implicating T-cell dysregulation in neurological disorders highlights the therapeutic feasibility and adaptability of brain T-cell populations, rendering them attractive targets for a wide spectrum of these diseases [53]. For instance, Treg-deficient mice exhibit significant impairments in remyelination and oligodendrocyte differentiation, experimentally demonstrating previously unrecognized regenerative functions of Tregs [54]. Moreover, TH17 cells play a critical pathogenic role in the nervous system. Studies demonstrate that TH17 cells sense danger signals via the C-type lectin receptor Mincle, thereby promoting the development of central nervous system inflammation. In the present study, histopathological analysis showed that SI alleviated tissue alterations in the hippocampus, thymus, and spleen (Figure 2F–H), and flow cytometry showed that SI was associated with partial normalization of CD4+ and CD8+ T-cell proportions in mesenteric lymph nodes and spleen. Together, these findings support the involvement of immune-related mechanisms in the effects of SI. Because H&E staining does not specifically assess microglial activation, the present interpretation of neuroinflammation relies mainly on brain transcriptomic findings and warrants further validation with Iba1 or other specific microglial markers. In addition, the current data do not establish a direct causal link between these immune-related changes and behavioral improvement, and the underlying mechanisms remain to be further clarified.

The gut–brain axis, a bidirectional communication network linking intestinal microecology to central nervous system function, has emerged as a critical regulatory pathway in neurodevelopmental disorders like ADHD [55]. Accumulating evidence suggests that gut dysbiosis may influence brain function through immune signaling, microbial metabolites, and barrier-related mechanisms. Research indicates that probiotics such as *Lactobacillus rhamnosus GG* may enhance the quality of life in ADHD patients by reducing levels of inflammatory cytokines [56]; early intervention could potentially lower the risk of ADHD development [57]. In the present study, 16S rRNA sequencing showed that SI treatment was associated with partial remodeling of gut microbiota dysbiosis in the model group, as reflected by changes in microbial diversity, community structure, and the relative abundance of specific taxa. However, the lack of direct evidence from blood and brain samples for microbial translocation or microbiota-derived components remains a limitation of the present study. Plasma metabolomics further indicated that SI treatment was associated with metabolic alterations in the scopolamine-induced ADHD-like phenotypes. Differential metabolites included linoleic acid and metyrosine, suggesting changes in lipid and amino acid metabolism (Figure 6G). Pathway analysis indicated enrichment in processes related to RORA signaling, fatty acid metabolism, and mitochondrial function (Figure 6H). Notably, we also observed differential metabolites associated with mitochondrial function, suggesting that SI may exert its effects partly through modulation of mitochondrial pathways. However, this possibility still requires further functional validation through mitochondria-related functional experiments. These metabolic changes collectively suppress excessive immune activation, reduce neuroinflammation, enhance neuronal protection, and may ultimately alleviate ADHD-like behavioral symptoms [33,58].

We also investigated the major ADHD-related components identified in SI to explore their potential pharmacological basis. UPLC-Q-Exactive Plus MS analysis identified 56 components in SI, with the top 10 ADHD-associated components including Oleamide, Tyramine, and multiple amino acids (Table 1). This compositional diversity suggests that the biological effects of SI may arise from multiple components. The presence of the inhibitory neurotransmitter GABA (Gamma-Aminobutyric Acid) and its precursor glutamic acid suggests possible relevance to excitatory/inhibitory balance [59,60], while tryptophan and tyrosine may be relevant to monoamine biosynthesis [61,62]. In addition, melatonin and caffeic acid have been reported to exhibit anti-inflammatory or neuroprotective activities [63,64]. In addition, SI also contains abundant inorganic elements, which are potentially relevant because metal elements are known to participate in immune regulation. Nevertheless, it must be emphasized that important aspects regarding these complex components in SI, such as bioavailability in humans, ability to effectively cross the blood–brain barrier, and the specific nature of their interactions, remain unclear. Therefore, the exact contribution and potential synergies of individual components within the complex SI matrix require further investigation.

Although the present study showed that SI was associated with attenuation of scopolamine-induced ADHD-like phenotypes, several limitations should be noted. The scopolamine-induced mouse model captures only selected behavioral and neurobiological abnormalities and does not fully recapitulate the complexity of ADHD. In particular, it is more closely related to cholinergic dysfunction than to the full pathophysiology of the disorder. Accordingly, future studies should incorporate more pathophysiologically relevant models, such as spontaneously hypertensive rats (SHR) and genetic ADHD models with stronger construct validity [65]. In addition, the behavioral assessment in the present study was limited mainly to spontaneous activity and the Morris water maze, which primarily reflect hyperactivity-like behavior and spatial memory-related changes rather than core ADHD dimensions such as attention deficits and impulsivity. The current design also does not distinguish among preventive, immediate blocking, and restorative effects of SI, because the intervention window was not structured for that purpose. Future work should therefore include different treatment schedules and co-administration paradigms to better define the stage-specific effects of SI. Mechanistically, the absence of mAChR-related pharmacological tools prevents further evaluation of whether SI directly affects cholinergic signaling, and whether SI alters the pharmacokinetics of scopolamine also remains unclear. In addition, the lack of direct evidence from blood and brain samples for microbial translocation or microbiota-derived components should be acknowledged as a further limitation. Since only male mice were included, the generalizability of the findings may also be limited in view of known sex differences in ADHD-related phenotypes and treatment responses. Finally, because ADHD is closely associated with dysregulation in dopamine- and norepinephrine-related signaling, the possible involvement of monoaminergic pathways in the effects of SI cannot be excluded and should be clarified in future studies. Together, these findings suggest that SI may serve as a multi-target intervention in a scopolamine-induced ADHD-like model, while further studies are needed to clarify its mechanisms and translational significance.

## 5. Conclusions

Through an integrative approach combining UPLC-Q-Exactive Plus MS component identification, behavioral assessment, histopathological analysis, flow cytometry, and multi-omics profiling, the present study showed that SI was associated with improvement of ADHD-like behavioral abnormalities in a scopolamine-induced model. These effects may be related to the anti-inflammatory and immunomodulatory properties of SI, as well as to changes in microbial diversity, specific bacterial taxa, metabolic pathways, and neuroinflammation-related processes. Collectively, these findings provide preliminary experimental evidence that SI has beneficial effects in a scopolamine-induced model showing ADHD-relevant phenotypes. Further mechanistic studies and, ultimately, clinical investigations will be needed to clarify its biological effects, translational relevance, and safety in humans.

## Figures and Tables

**Table 1:** Top 10 active ingredients of SI and their associated targets for ADHD.

Compound Name	Rt (min)	Experimental *m/z*	Adduct Type	Formula	Class	ADHD-Related Targets
Glutamic acid	0.904	148.06061	[M+H]^+^	C_5_H_9_NO_4_	Carboxylic acids and derivatives	GRIA1; SLC1A1; GRIK2; GRM1; GRIA2
Phenylalanine	6.045	164.07045	[M−H]^−^	C_9_H_11_NO_2_	Carboxylic acids and derivatives	GABBR2; GABBR1; TH; SLC6A4; TAAR1
Oleamide	11.832	282.27884	[M+H]^+^	C_18_H_35_NO	Fatty Acyls	FAAH; CYP19A1; CES1; MAPK14
Tyramine	3.864	138.09142	[M+H]^+^	C_8_H_11_NO	Benzene and substituted derivatives	TAAR1; DRD2; SLC6A3
Octopamine	2.157	154.08609	[M+H]^+^	C_8_H_11_NO_2_	Phenols	ADRB2; DRD2; OPRM1
Arginine	0.862	175.11911	[M+H]^+^	C_6_H_14_N_4_O_2_	Carboxylic acids and derivatives	NOS3; NOS1
Citrulline	0.932	176.10283	[M+H]^+^	C_6_H_13_N_3_O_3_	Carboxylic acids and derivatives	NOS3; NOS1
Valine	6.66	118.08664	[M+H]^+^	C_5_H_11_NO_2_	Carboxylic acids and derivatives	GABBR2; GABBR1
GABA	0.932	104.07121	[M+H]^+^	C_4_H_9_NO_2_	Carboxylic acids and derivatives	GABBR2; GABBR1
Cinnamic acid	6.02	149.05989	[M+H]^+^	C_9_H_8_O_2_	Cinnamic acids and derivatives	HCAR2

Abbreviations for gene symbols follow standard HGNC nomenclature. Rt: Retention time; *m*/*z*: Mass-to-charge ratio.

**Table 2:** Quantitative results of inorganic elements in SI.

Component	Content (Mean ± SEM)
Ca	11,566.67 ± 622.72 mg/kg
Mg	11,833.33 ± 841.30 mg/kg
Mn	3.25 ± 0.06 mg/kg
Fe	36.83 ± 0.50 mg/kg
Zn	88.80 ± 9.04 mg/kg
Se	5.00 ± 0.64 mg/kg

## Data Availability

The data are not publicly available due to privacy restrictions.

## Supplementary Materials

The following supporting information can be downloaded at: https://www.mdpi.com/article/10.3390/cimb48040410/s1.

## Abbreviations

The following abbreviations are used in this manuscript:

ANOSIM	Analysis of Similarities
APC	Allophycocyanin
ASV	Amplicon Sequence Variants
BDNF	Brain-Derived Neurotrophic Factor
CMC-Na	Carboxymethyl Cellulose Sodium
CNS	Central Nervous System
COX-2	Cyclooxygenase-2
CREB1	cAMP Responsive Element Binding Protein 1
DEGs	Differentially Expressed Genes
ESI	Electrospray Ionization
EOs	Eosinophils
FAM	Fatty Acid Amide
FITC	Fluorescein Isothiocyanate
GSEA	Gene Set Enrichment Analysis
GO	Gene Ontology
H&E	Hematoxylin and Eosin
HCT	Hematocrit
ICP-MS	Inductively Coupled Plasma Mass Spectrometry
ICP-OES	Inductively Coupled Plasma Optical Emission Spectrometry
IFN-γ	Interferon-gamma
IFN-α/IFN-β	Interferon-alpha/Interferon-beta
IAV	Influenza A virus
IL-6	Interleukin-6
iNOS	inducible Nitric Oxide Synthase
IPA	Ingenuity Pathway Analysis
JAK-STAT	Janus Kinase-Signal Transducer and Activator of Transcription
KEGG	Kyoto Encyclopedia of Genes and Genomes
LDA	Linear Discriminant Analysis
LEfSe	Linear Discriminant Analysis Effect Size
LFA-1	Lymphocyte Function-associated Antigen 1
LPS	Lipopolysaccharide
mAChR	Muscarinic Acetylcholine Receptor
MAPKs	Mitogen-Activated Protein Kinases
MAOA	Monoamine Oxidase A
M1/M2	Macrophage Type 1/2
MLNs	Mesenteric Lymph Nodes
MUFA	Monounsaturated Fatty Acid
NF-κB	Nuclear Factor-κB
NMDS	Non-metric Multidimensional Scaling
OPLS-DA	Orthogonal Partial Least Squares-Discriminant Analysis
OTUs	Operational Taxonomic Units
PCoA	Principal Coordinates Analysis
PE/Cyanine7	Phycoerythrin/Cyanine7
PI3K-Akt	Phosphatidylinositol 3-Kinase-Akt
PPI	Protein–Protein Interaction
PROTAC	Proteolysis Targeting Chimera
QC	Quality Control
RBC	Red blood cells
RORA	Retinoic Acid-Related Orphan Receptor Alpha
SCFA	Short-Chain Fatty Acid
SHR	Spontaneously Hypertensive Rat
SLC6A3	Solute Carrier Family 6 Member 3
Th1/Th2/Th17	T Helper Cell 1/2/17
TNF-α	Tumor Necrosis Factor-alpha
Treg	Regulatory T Cell
UHPLC	Ultra-High-Performance Liquid Chromatography
WBC	White blood cells
