# Supplementary material for: Integrative Multi-Omics Analysis Reveals the Immunoregulatory Effects of Sepia Ink on ADHD-like Phenotypes
Source: Curr Issues Mol Biol. 2026 Apr 16;48(4):410. doi: 10.3390/cimb48040410 (PMC13114676; doi:10.3390/cimb48040410)
Supplement: Supplementary file 1 [file cimb-48-00410-s001.zip › cimb-4188861-supplementary.pdf]

Integrative Multi-Omics Analysis Reveals the Immunoregulatory Effects of Sepia Ink on ADHD-Like Phenotypes

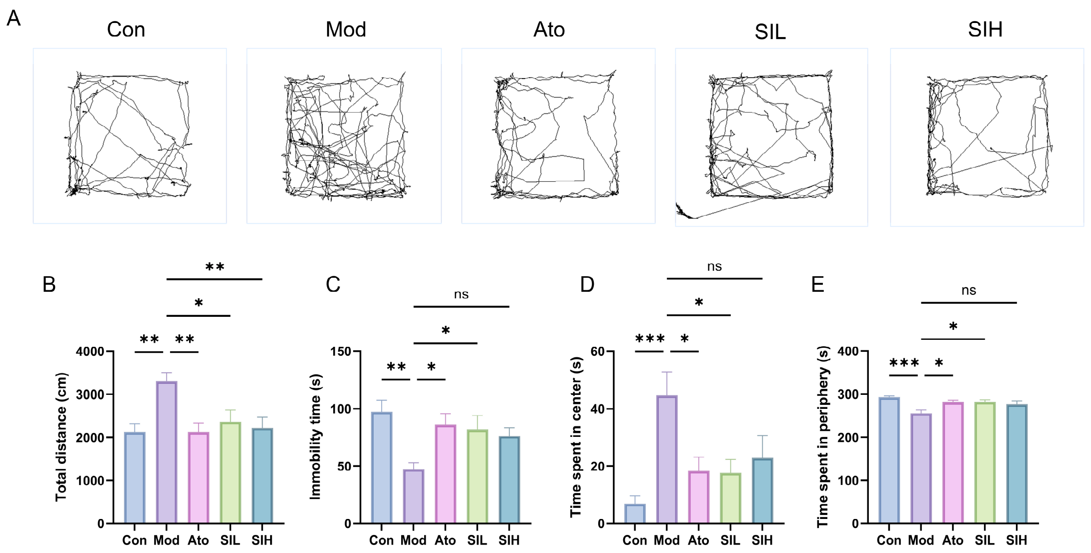

Figure S1. SI ameliorated hyperactivity-like behavior in SHR rats in the open-field test.
(A) Representative locomotor trajectories of rats in the Con, Mod, Ato, SIL, and SIH groups. (B) Total distance traveled. (C) Immobility time. (D) Time spent in the center. (E) Time spent in the periphery. Data are presented as the mean ± SEM (n = 8). $^{*}P < 0.05$, $^{**}P < 0.01$, $^{***}P < 0.001$; ns, not significant.

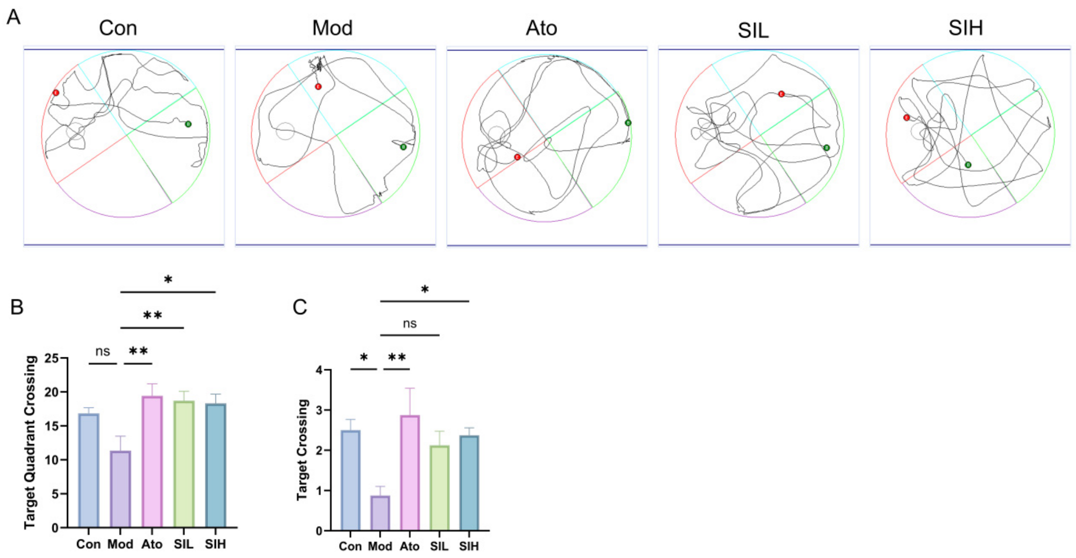

Figure S2. SI improved spatial memory performance in SHR rats in the Morris water maze test.

(A) Representative swimming trajectories of rats in the probe trial in each group. (B) Target quadrant crossing. (C) Target crossing. Data are presented as the mean ± SEM (n = 8). $^{*}P < 0.05$, $^{**}P < 0.01$; ns, not significant.

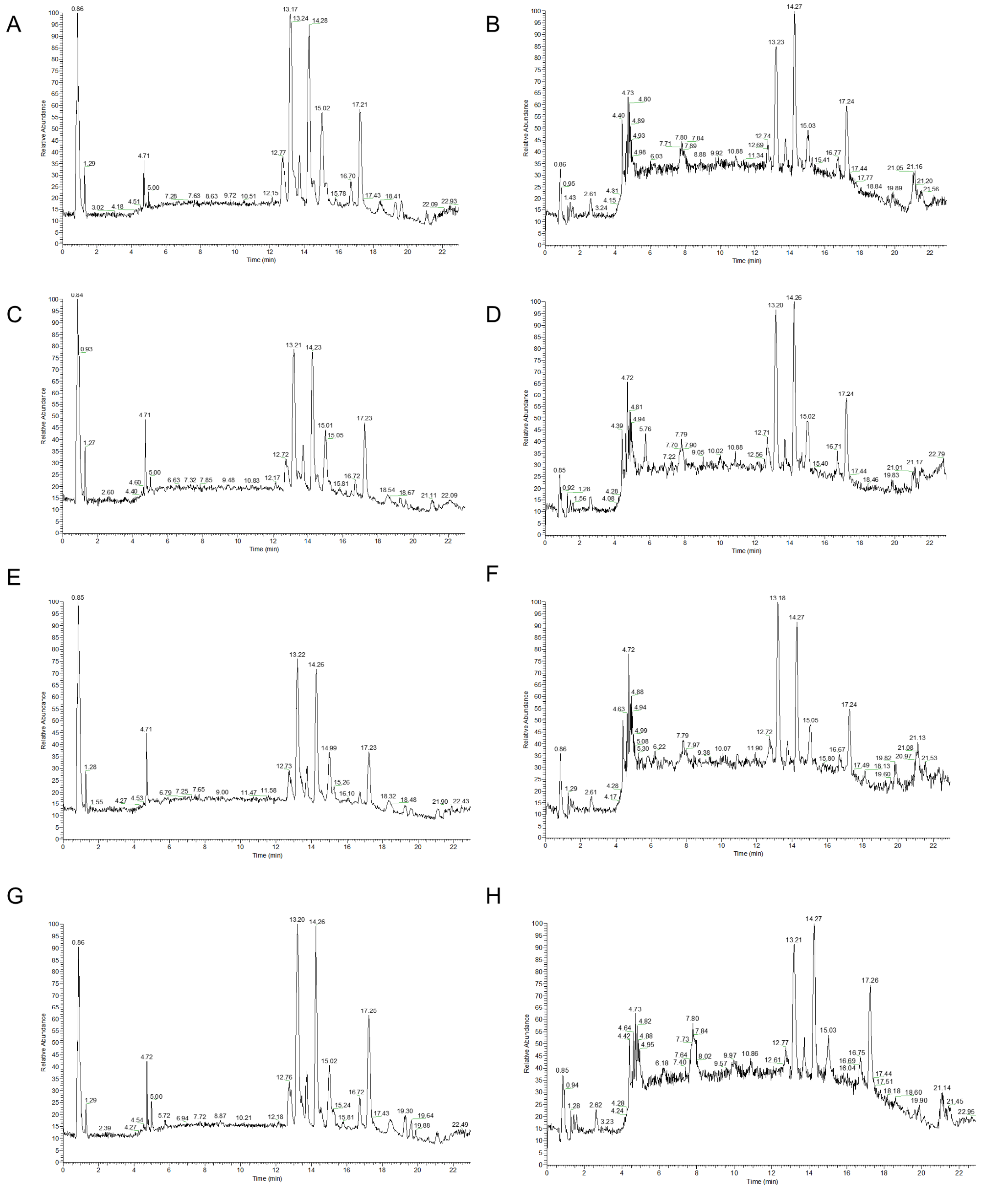

Figure S3. Multivariate analysis of plasma metabolomic profiles in negative and positive ion

modes.

(A) Con in negative ion mode. (B) Con in positive ion mode. (C)Mod in negative ion mode. (D) Mod in positive ion mode. (E) SIL in negative ion mode. (F) SIL in positive ion mode. (G) SIH in negative ion mode. (H) SIH in positive ion mode.
